# Update on *CECR1* molecular diagnostics: new mutations in the deficiency of ADA2 (DADA2) and the North American polyarteritis nodosa (PAN) cohort

**DOI:** 10.1186/1546-0096-13-S1-O20

**Published:** 2015-09-28

**Authors:** M Stoffels, Q Zhou, C Chen, I Aksentijevich, DL Kastner, PA Merkel, the Vasculitis Clinical Research Consortium

**Affiliations:** 1 National Institutes of Health, National Human Genome Research Institute, Bethesda, USA; 2 University of Pennsylvania Perelman School of Medicine, Division of Rheumatology, Philadelphia, USA; 3 University of Pennsylvania, Philadelphia, USA

## Background

Loss-of-function mutations in *CECR1*, encoding adenosine deaminase-2 (ADA2), have been associated with a spectrum of vascular and inflammatory phenotypes, ranging from early-onset vasculopathy manifesting as recurrent stroke, to systemic vasculitis manifesting as polyarteritis nodosa (PAN) and Sneddon's Syndrome.

## Objectives

Knowledge about the ADA2 genotype and phenotype has only recently started to emerge. We aimed to further characterize the genotype-phenotype spectrum associated with *CECR1* mutations.

## Patients and methods

19 newly diagnosed DADA2 patients and 92 DNA samples of the North American cohort with PAN were screened for mutations in *CECR1* by Sanger sequencing of all coding exons.

## Results

14/19 patients were homozygous or compound heterozygous for rare or novel missense mutations in the coding region of *CECR1*. In 5 patients, we have detected only one likely disease-associated mutation; further analysis is necessary to identify a possible genomic deletion or a second mutation in the non-coding region of *CECR1*. We have found three rare missense mutations that were not previously associated with DADA2: A357T, G358R, and L249P. More importantly, we have identified four novel mutations that cause DADA2: T129P, K55del, N370K and N423K. The R169Q mutation is a founder mutation in the Dutch population, while the G47R mutation is a founder mutation in the Middle Eastern and Pakistani populations.

In the PAN cohort, we identified 6/92 patients with mutations in *CECR1*. Three patients carry biallelic homozygous or compound heterozygous mutations, and three patients are carriers for a single mutation in *CECR1*. Four rare variants are reported in Ensemble or ExAC, but they have not been previously associated with PAN: P106S, F355L, V349I, and T65M. We have identified one novel mutation in the cohort with PAN: E328K.

## Conclusion

The *CECR1* gene is highly polymorphic, and interpreting identified gene variants should be done cautiously. When possible, parental samples should be used to demonstrate proper inheritance of biallelic variants. Biochemical assays may help to complement molecular diagnostics. We have identified a significant number of patients who carry only a single novel or rare mutation in *CECR1.*These patients should be analyzed for the presence of structural or non-coding variants in *CECR1*. Alternatively, we will consider the possibility that single mutations may act as susceptibility alleles for complex forms of vasculitis. Our study expands on the role of ADA2 in the pathogenesis of PAN in non-founder populations.

